# Effect of undersized drilling on the stability of immediate tapered implants in the anterior maxillary sector. A randomized clinical trial

**DOI:** 10.4317/medoral.24107

**Published:** 2021-01-04

**Authors:** Alejandro Sierra-Rebolledo, Dayana Tariba-Forero, Maria del Pilar Rios-Calvo, Cosme Gay-Escoda

**Affiliations:** 1 DDS, MSc. Associate Professor of Oral Surgery and Implantology and Coordinator of the Postgraduate Program in Dental Implantology. University of Carabobo, Dental School, Valencia, Venezuela; 2 DDS, MSc. Master Degree in Dentistry and Dental Implantology, Santa María University, Dental School, Caracas, Venezuela; 3 DDS, MSc. Associate Professor and Director of the Master Degree in Dentistry and Dental Implantology, Santa María University, Dental School, Caracas, Venezuela; 4 MD, DDS, MSc, PhD, EBOS, OMFS. Chairman and Professor of Oral and Maxillofacial Surgery. Head of the Department of Oral and Maxillofacial Surgery, University of Barcelona. Coordinator of the Dental Therapy and Maxillofacial Diseases Research Group (IDIBELL). Head of the Department of Oral and Maxillofacial Surgery and Implantology, Teknon Medical Center, Barcelona, Spain

## Abstract

**Background:**

To evaluate the effect of undersized drilling on the primary and secondary stability of immediate implants placed in the anterior maxilla.

**Material and Methods:**

A comparative randomized clinical trial was carried out in 30 healthy adults. Thirty tapered implants, 16 involving conventional drilling and 14 undersized drilling, were placed immediately after anterior maxillary tooth removal. Insertion torque and implant stability assessed by resonance frequency analysis (RFA) were evaluated at three different timepoints: at implant placement and 6 and 12 weeks post-implantation. The results were compared using parametric statistical tests.

**Results:**

All implants showed adequate stability during follow-up. At implant placement, the undersized drilling group exhibited greater insertion torque values than the conventional drilling group, but stability assessed by RFA showed greater mean values in the conventional group. After 6 and 12 weeks of follow-up, both groups showed improved stability, though the RFA values remained comparatively higher in the conventional group. The differences were not statistically significant.

**Conclusions:**

Based on the results obtained, undersized drilling does not appear to afford significantly improved stability of immediate implants placed in the anterior zone of the maxilla during the osseointegration period.

** Key words:**Insertion torque, RFA, undersized drilling, immediate implants, primary stability, secondary stability.

## Introduction

Peri-implant tissue preservation is crucial when planning restorations on implants placed in aesthetics areas such as the anterior sector of the maxilla. As is well known, tooth extraction is followed by progressive loss of height and thickness of the alveolar bone walls (1,2). Specifically, in the anterior sector of the maxilla, a decrease of about 37% in alveolar bone volume has been reported one year after tooth extraction (3).

Immediate implant placement has been proposed as a way to avoid soft and hard tissue dimensional loss (4-6). However, this way of placing implants in aesthetically sensitive sectors requires tooth extraction with minimum trauma to both the soft tissues and the bone surrounding the implants. Furthermore, the surgeon must be able to place the implants in circumstances of limited available bone, reaching primary stability conditions equal to or better than 35 Ncm of insertion torque (IT) or an implant stability (IS) of 65 ISQ determined by resonance frequency analysis (RFA)(7,8).

Modifications in drilling speed or diameter have been suggested with the purpose of ensuring primary stability parameters capable of guaranteeing osseointegration under conditions characterized by deficient bone quantity and density. Specifically, reduction of the final diameter of the implant bone bed by means of an incomplete drilling sequence known as “undersized drilling” has been proposed to increase IT when placing implants in bone of deficient quality or quantity (9). Studies in animal models have biomechanically and histologically evaluated the effect of undersized drilling upon implant healing. The results reflect an increase in IT as the final drilling diameter is reduced. However, at histological level, no statistically significant differences were observed in terms of bone-implant contact (BIC) at the end of the osseointegration period. Furthermore, a variable healing pattern was recorded, capable of affecting implant stability during the period between 3-6 weeks after placement (10-13).

To date, few data have been published on the benefits of undersized drilling. The technique has been regarded as safe in cases characterized by poor bone density, with no significant differences in percentage osseointegration versus conventional drilling (14). Nevertheless, comparatively greater cortical bone loss at cervical level has been reported with the undersized drilling approach (14,15).

Limited information is available on the effects of undersized drilling in cases of immediate or early implant placement. A follow-up study based on tomographic imaging of three immediate implants showed no variations in bone quality beyond 6 months after the immediate placement of implants with an undersized drilling discrepancy of 28% in the anterior maxilla. However, the authors underscored the need to investigate bone behavior during the osseointegration period, since undersized drilling apparently affects percentage remodeling around the implants (16). A previous study on the effect of four different undersized drilling protocols under conditions similar to those of immediate implant placement in a model of human bone analogs with different density types recorded an increase in IT and ISQ determined by RFA as the final drilling diameter was reduced. However, the differences among the protocols were not statistically significant, and the authors moreover concluded that primary stability under conditions of immediate implant placement could be more associated to bone density than to the drilling protocol used (17).

Based on the above, and due the lack of clinical studies referred to undersized drilling with immediate implant placement, a comparative clinical study has carried out to evaluate the effect of undersized drilling on the primary and secondary stability of immediate implants placed in the anterior sector of the maxilla.

## Material and Methods

- Study design

A randomized, comparative parallel-group clinical trial was carried out in a series of adults requiring single implant treatment and the extraction of a maxillary anterior tooth. The patients were selected according to the enrollment criteria of the population seen in the clinic of the Master of Dentistry and Dental Implantology (Santa Maria University, Dental School, Caracas, Venezuela). The study was approved by the Bioethics Committee of Santa Maria University Dental School (Ref. CBB-FO-USM31032015), and the protocol was registered with ClinicalTrials.gov number NCT04345133.

- Patient selection 

The patients were required to be over 18 years of age, with no history of ischemic heart disease, uncontrolled diabetes, coagulation disorders, head or neck radiotherapy, intravenous bisphosphonate use or uncontrolled periodontal disease. Furthermore, cone-beam computed tomography (CTCB) was required to confirm the presence of bone ≥ 5 mm from the tooth apex to the lower cortical layer of the nasal fossa / maxillary sinus, with no vertical defects greater than 4 mm at the buccal or palatine alveolar crest. Also, the included patients were required to be able to understand the study protocol and give written informed consent to participation in the study.

- Implant designs and features

Thirty tapered conical connection grade 23 titanium alloy (Ti 6Al 4V Eli) implants with a dual acid etching and sandblasting rough surface, and measuring 3.75 mm in diameter and 13 mm in length (C1 Implants, MIS® Implants Technologies Ltd., Bar Levi, Israel) were used in all cases.

- Interventions procedure

Two operators calibrated for immediate implant placement performed minimally traumatic tooth extraction under local anesthesia with 4% articaine and 1:100,000 epinephrine (Artheek® 4%, New Stetic S.A., Antioquia, Colombia). A periodontal probe was used to evaluate the integrity of the bone walls and thus decide whether the minimum bone height required for implant placement was present.

- Sample size

Sample size calculation was performed to establish the minimum number of subjects to be included in the study. The criterion for significance was established as 5% for type I error and 10% for type II error. Taking into consideration a minimum expected effect size in ISQ values of 7 with a standard deviation (SD) of 5 (17), and assuming a 15% dropout rate, a minimum of 28 subjects (14 per group) were required.

- Random group assignment 

The patients were assigned to two groups through simple randomization with 1:1 allocation ratio. An external observer tossed a coin just before the start of the drilling sequence and thus assigned the patient to the corresponding group. The drilling sequence was performed following the drilling protocol recommended by the manufacturer in the case of poor bone density.

A) Conventional drilling sequence group (CD): complete drilling protocol following the sequence: 1) Marking drill ∅1.9 mm at 1500 rpm; 2) Pilot drill ∅2.4 mm and 13 mm in length at 800 rpm; and finally, 3) Twist drill ∅3 mm at 400 rpm.

B) Undersized drilling sequence group (UD): undersized drilling protocol following the sequence: 1) Marking drill ∅1.9 mm at 1500 rpm; and 2) Pilot drill ∅2.4 mm and 13 mm in length at 400 rpm.

Particular care was taken to maintain blinding of the participants and in data collector during follow-up of the implants.

- Implant insertion

A surgical motor (MCU MIS, model M0132, W&H, Burmoos, Austria) with a 20:1 reducing implant handpiece was used to insert the implants at 20 rpm speed and 10 Ncm torque. The final position of the implants inside the socket was achieved with a ratchet, up to a depth of 4 mm from the gingival margin.

- Study variables

Insertion torque: Two torque meters (MIS® Implants LTD, models MT-RI040 and MT-RT070, Bar Levi, Israel) were used to insert and sequentially measure the maximum IT reached on positioning the implant in the socket.

Implant stability assessed by RFA: A Smart-peg Nro 49 model 100480 was fitted to the connection of each implant, and an Osstell ISQ® (SN 4669 Osstell AB, Goteborg, Sweden) was used to perform RFA analysis and obtain the corresponding ISQ value at three different timepoints during implant osseointegration: at insertion (RFA1), at 6 weeks (RFA2) and at 12 weeks (RFA3) post-implantation. A 4 mm height healing screw was used to seal the implant and thus allow access for measurements.

- Statistical analysis

The SPSS version 20.0 statistical package was used to compare the data obtained in both groups. The Kolmogorov-Smirnov test was used to assess normal data distribution. The Student t-test was used to compare the mean IT values, and analysis of variance (ANOVA) for repeated measures was used to compare ISQ values of groups at the three timepoints. Statistical significance was considered for *p* < 0.05.

## Results

**Figure 1:**
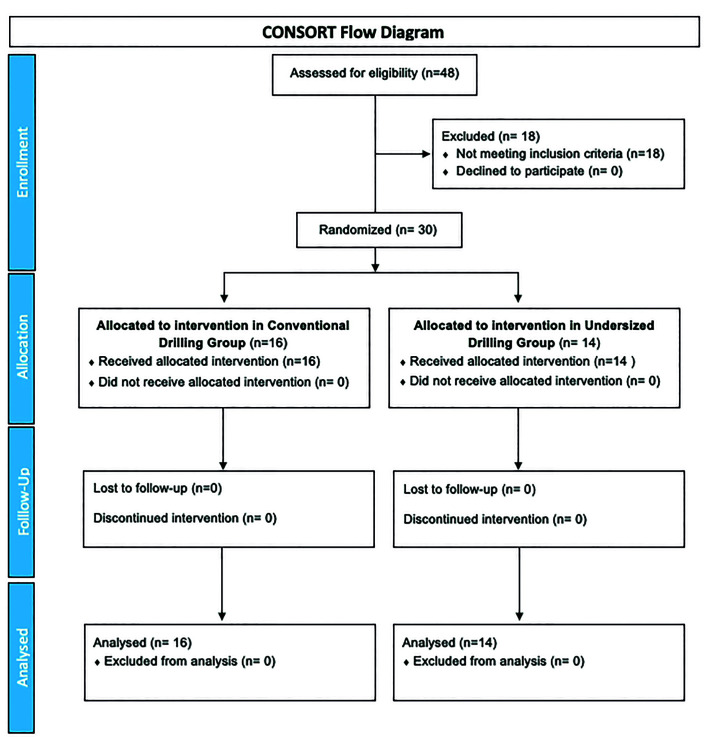
CONSORT flowchart.

Thirty patients, 19 females (63.3%) and 11 males (36.7%), with a mean age of 48.87 ± 14.84 years, were included in the study and 30 tapered implants - 16 belonging to the CD group (53.3%) and 14 belonging to the UD group (46.7%) - were successfully placed immediately after tooth extraction in the anterior maxilla. The demographic characteristics and implant placement zone are reported in Table 1. No adverse events or implant failures were recorded during the 12 weeks of follow-up. The study flowchart based on the CONSORT statement is shown in Fig. 1.

- Insertion torque

The median IT of the overall implants was 35 Ncm, with a mean of 39.80 ± 7.14 Ncm (Table 2). The mean IT was higher in the UD group (41.36 ± 18.86 Ncm) than in the CD group (38.44 ± 15.99 Ncm), though the difference failed to reach statistical significance (*p* = 0.654) (Table 3).

- Implant stability assessed by RFA

A progressive and similar increase in implant stability was observed over the weeks following implant placement in all cases. The ISQ values during the study ranged from 50.0-79.5 (Table 2). Comparison of the mean ISQ values (RFA) using the Student t-test showed no statistically significant differences between the groups at the three measurement timepoints. However, the CD group always showed higher ISQ values than the UD group (Table 4).

**Figure 2:**
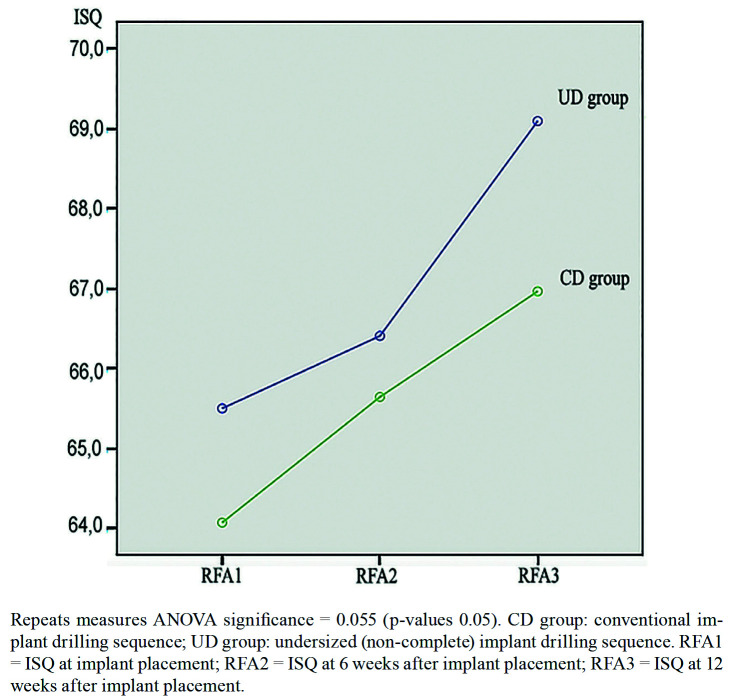
Evolution of implant stability assessed by RFA according to estimated marginal means of ISQ at the three evaluation timepoints.

Analysis of variance for repeated measures was used to compare implant stability, defined as implant immobility assessed by RFA at the three measurement timepoints (at implantation and 6 and 12 weeks post-implantation), and showed a progressive and similar increase in ISQ values over the weeks in both groups - with no significant differences between them (Fig. 2).

**Table 1:**
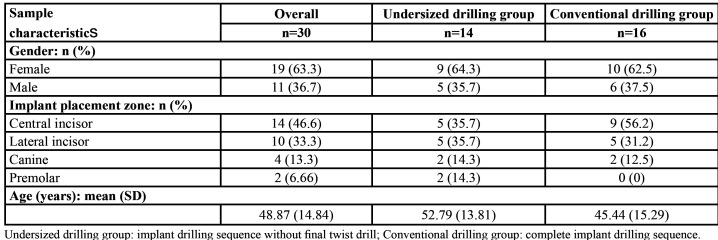
Distribution of the patients according to gender, implant placement zone and age.

**Table 2:**
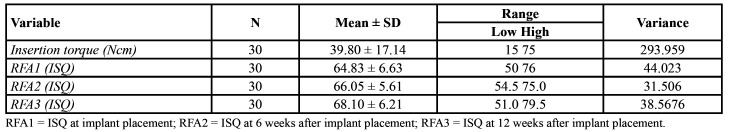
Insertion torque and implant stability assessed by RFA (ISQ) of the overall implants at insertion.

**Table 3:**
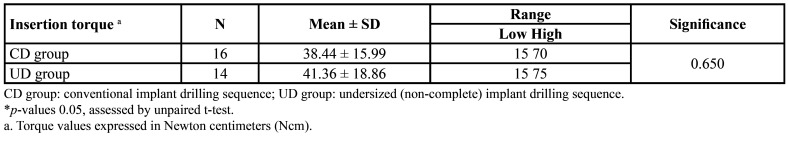
Comparison of insertion torque between the conventional drilling (CD) and undersized drilling (UD) groups at implant placement time.

**Table 4:**
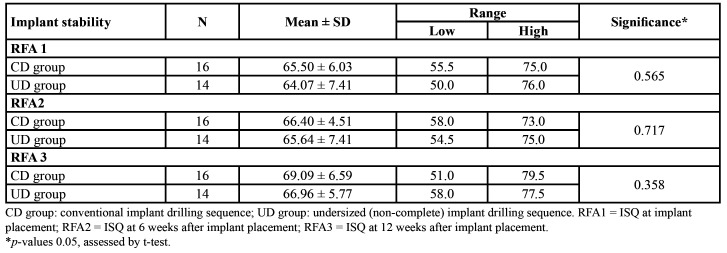
Comparison of implant stability assessed by RFA (ISQ) between the groups at the three evaluation timepoints.

## Discussion

Modification of the drilling sequence in order to increase the primary stability of implants placed in poor density bone was first proposed by Friberg *et al*. in 1999 (9). However, it was not until the beginning of this last decade that interest appeared in investigating the effect of reducing the drilling diameter upon primary stability and peri-implant bone behavior during the healing period. Experimental studies in biomechanical analogs of bone, animal models and cadaveric human bone have demonstrated an inversely proportional relationship between reduced final drilling diameter and primary implant stability assessed on the basis of insertion torque (IT) or resonance frequency analysis (RFA) (10,11,13,17,18).

Very few clinical studies have evaluated the evolution of implant stability after modifying the final drilling diameter (19,20). Gonzalez-Martin *et al*. (16) reported IT values suggesting the technique to be feasible. The present study is probably the first randomized comparative clinical trial to assess the effect of undersized drilling upon the primary stability of immediate implants. The IT (mean: 39.80 ± 7.14, range: 17-75 Ncm) and implant stability assessed by RFA (mean: 64.83 ± 6.63, range: 50-70 ISQ) for the overall implants recorded at implant placement confirmed the viability of the immediate implantation technique, with sufficient mechanical stability to ensure osseointegration. The mean values were comparable to those reported by Levin (IT: 28 Ncm and RFA: 68 ISQ) placing 59 tapered implants of different lengths in the anterior sector of the maxilla (21). In contrast, the IT values were higher than those obtained by Degidi *et al*. (22) on evaluating 606 tapered implants placed in the maxilla (33.56 Ncm). These discordant results between studies could be explained by differences in the implant apex design, drilling protocol and twist drill diameters. The RFA values for the global sample at the time of implant insertion were similar to those found in other studies involving different bone volume conditions (22-25).

We have found no human clinical studies with a similar design comparing the effects of undersized drilling versus conventional drilling upon the stability of immediate implants. The comparative analysis of the sample confirmed that reducing the diameter of drilling increases the IT values (41.36 ± 18.86 Ncm in the UD group versus 38.44 ± 15.99 Ncm in the CD group). These results are similar to those of previous studies by our group conducted in analog of human bone (17) and by Coelho *et al*., involving similar designs but conducted in animal models, where undersized drilling was seen to be associated to increased IT - though no differences were found in terms of bone-implant contact (BIC) or bone area fraction occupied (BAFO), as assessed by histological analysis (11,13). In a clinical study of 108 implants in fully healed bone, Toia *et al*., evaluated the effects of different drilling protocols with or without countersink drills selected according to clinician criterion during osteotomy. The results confirmed a difference in IT between the undersized drilling model and conventional drilling, particularly under conditions of low bone density (15). However, and in coincidence with the observations in the literature, the IT increments recorded with undersized drilling failed to reach statistical significance.

Several publications have suggested that there is no clear association between IT and the RFA findings on assessing mechanical stability at the time of implant placement (21,22,26,27). The results of our study support this conclusion, since the mean RFA in the CD group, where IT was lower at the time of insertion, was greater than in the UD group (65.50 ± 6.03 versus 64.07±7.41, respectively) - though the difference was not statistically significant. Apparently, increasing the IT values does not necessarily imply an increase in stability as assessed by RFA.

Greater IT values associated to undersized drilling have been attributed to higher bone compression, and therefore to greater bone remodeling during osseointegration, which theoretically could affect implant stability during this period (10,11,14). Kim *et al*. used RFA in the first 10 weeks after inserting 25 implants with different diameters and surfaces, and recorded a decrease in ISQ values during the first three weeks after implant placement in mature bone, followed by a recovery of these values from the fourth week and a gradual increase in stability as assessed by RFA from the sixth to the twelfth week (19). The present study also appears to be the first to follow-up on the stability of tapered implants placed immediately after tooth extraction, and in agreement with the findings of Kim *et al*. (19), after week 6 of follow-up higher mean ISQ values were recorded in both groups versus those recorded at the time of implant placement.

Throughout follow-up of the implants, the CD group presented greater stability as assessed by RFA than the UD group. These results could support the observations of Coelho *et al*. and Norton, who suggested that increased implant IT could cause a loss of stability in the early stages of osseointegration due to increased bone remodeling produced by bone compression and warming secondary to friction caused by the implant upon insertion (11,28,29). It would be relevant to evaluate the behavior of the RFA findings during the first four weeks of osseointegration, since this appears to be the period in which greater changes in implant stability may be observed. In this regard, the secondary stability reached by week 6 seems to suffice to guarantee successful osseointegration.

Twelve weeks after placement of the tapered implants, the mean ISQ values corresponding to the overall sample were seen to continue to increase, reaching 68.10 ± 6.21. In the same way as at the previous timepoints, the mean ISQ values were lower in the UD group than in the CD group, and although the differences were not statistically significant, from the clinical perspective it could be assumed that undersized drilling during immediate implant placement in the maxilla was not necessary.

The mean RFA values obtained during follow-up of the immediate implants in the anterior maxilla did not reach ISQ 70, which is defined as optimum for immediate occlusal loading. Therefore, within the limitations and under the conditions of the present study, this could imply that regardless of the IT values obtained at insertion, occlusal loading of immediate implants in the anterior zone of the maxilla should take place after 12 weeks, when osseointegration has occurred.

## Conclusions

Based on the results obtained, undersized drilling does not appear to afford significantly improved stability of immediate implants placed in the anterior sector of the maxilla during the osseointegration period.
